# Interaction effect of midday napping duration and depressive symptoms on subjective memory impairment among older people in China: evidence from the China health and retirement longitudinal study database

**DOI:** 10.1186/s12889-023-16928-6

**Published:** 2023-10-13

**Authors:** Li Tang, Ya-qi Wang, Na-ni Zhan, Can-Yang Li, Zhuang Zhuang, Qi-yuan Lyu, Peng Xiong

**Affiliations:** 1 School of Nursing, Jinan University, Room 1015, Guangzhou, China; 2 Department of Public Health and Preventive Medicine, School of Medicine, Jinan University, 601 West Huangpu Road, Guangzhou, China

**Keywords:** Subjective memory impairment, Aged, Nap, Depression

## Abstract

**Background:**

Subjective memory impairment (SMI) is common in older people. The aim of this study was to investigate the factors influencing SMI among older people in China, with specific focus on the interaction effect of midday napping duration and depressive symptoms on the risk of SMI.

**Methods:**

Using a dataset representative of the Chinese population from a longitudinal study of health and retirement in China, subjects with SMI were screened using the question “how do you feel about your memory now?” and the Mini-Mental State Examination. A logistic regression model was applied to explore the factors affecting SMI. Additive and multiplicative models were used to analyze the interaction effect of midday napping duration and depressive symptoms on the risk of SMI.

**Results:**

We enrolled 8,254 subjects included and the incidence of SMI was 63.9%. Depressive symptoms, nap time, and physical activity were influencing factors of SMI. Midday napping duration and depressive symptoms had positive additive interaction effects on the risk of SMI. When extended-length naps and depressive symptoms coexisted, the risk of SMI was 1.06 times greater than that for either alone (RERI, relative excess risk due to interaction = 0.27, 95% CI = 0.07–0.43; AP, attributable proportion = 0.14, 95% CI = 0.01–0.23; S, synergy index = 1.06, 95% CI = 0.57–1.62). When short naps and depressive symptoms coexisted, the risk of SMI was 1.2 times higher than that for either alone (RERI = 0.12, 95% CI=-0.14–0.39; AP = 0.13, 95% CI=-0.07–0.22; S = 1.20, 95% CI = 0.79–1.82).

**Limitations:**

Since this was a cross-sectional study, the cause-and-effect relationships between the associated variables cannot be inferred.

**Conclusions:**

The interaction effect that exists between nap time and depressive symptoms in older people is important for the identification and early intervention of people at risk for SMI.

## Background

It is projected that the population of individuals aged ≥ 65 years in China will surge to 449 million by the year 2050, with the aging rate reaching an alarming 37.3% [1]. This demographic shift will inevitably lead to a substantial increase in the number of people afflicted by Alzheimer’s disease (AD) [2]. AD, being a neurodegenerative ailment, initiates through a continuous pathophysiological process that takes root before noticeable cognitive impairments manifest in AD patients. In the later stages of AD, a significant number of patients’ neurons undergo apoptosis, culminating in irreversible cognitive damage [3]. Owing to the inconspicuous nature of AD’s pathology progression, early identification and intervention become of paramount importance.

Subjective Memory Impairment (SMI) constitutes the prodromal phase of Mild Cognitive Impairment (MCI). SMI refers to self-reported memory decline observed in certain older people individuals, who nonetheless perform within the normal range on objective cognitive assessments [4]. The prevalence of SMI among older people ranges from approximately 20–70% worldwide [5–9]. The cognitive faculties of older people individuals with SMI deteriorate more rapidly compared to those without SMI, heightening the risk of developing AD within the former group [10, 11]. A 4-year cohort study further corroborated that 24.4% of older people exhibiting SMI eventually progressed to MCI [12]. Furthermore, older people with SMI are twice as likely to develop AD when compared to those without SMI [12]. Older people with SMI also experience abnormal neural activity in memory-related brain regions before the external memory behavior has been damaged [13].

Previous research has revealed a noteworthy correlation between the duration of midday napping and the memory proficiency of older people [14]. Interestingly, the percentage of older people who routinely indulge in midday naps stands between 22 and 69% globally, markedly surpassing the proportion observed among the younger population [15, 16]. Remarkably, a national cohort study conducted in China established that over 51% of older people adopt the practice of midday napping, considered culturally integral to a healthy lifestyle [17, 18]. Moreover, investigations have demonstrated a direct impact of midday napping duration on the escalation of β-amyloid deposition in the brain, which is intricately linked to an individual’s cognitive function [19, 20]. Notably, a large-scale study in China has directly ascertained that appropriately timed midday napping after lunch bestows a protective effect on the memory and cognitive function of older people [21]. As of now, numerous studies have delved into the causal association between nighttime sleep and SMI in older people. Many studies found that the mechanism by which nighttime sleep impairment affects the occurrence of SMI is related to β-amyloid deposition in the brain, daytime mental state, etc. [20, 22–24]. More research to clarify the relationship between midday napping duration and SMI remains necessary.

Depression constitutes a prevalent mental health challenge among older people, and the incidence of depressive symptoms in Chinese older people has witnessed an upward trend in recent times [25, 26]. A multitude of studies have substantiated the link between depressive symptoms and the manifestation of SMI [22, 27, 28]. For instance, Yates and colleagues reported that individuals exhibiting depressive symptoms displayed a heightened likelihood of developing SMI within two years [29]. Moreover, research has illuminated the association between depressive symptoms in couples and the onset of SMI in their later years [30]. However, investigations exploring the interplay of depression and midday napping on cognitive function have been scant.

Numerous scholars have delved into the correlation between the duration of midday napping, depressive symptoms, and SMI. However, the bulk of existing observational data stems from Western countries, and there is a dearth of comparable empirical research concerning older people in China. Variances in social and cultural backgrounds, lifestyle choices, and environmental factors may exert distinct influences on SMI among older people. Additionally, pivotal questions remain inadequately addressed: (1) whether an interaction effect exists between midday napping duration and depressive symptoms in older people, and (2) to what extent this effect is associated with SMI in older people. Regrettably, these topics have received insufficient exploration in the current literature. As a prodromal stage of MCI, SMI assumes a crucial role in predicting future cognitive decline and/or dementia in older people. Thus, in this study, we aimed to identify risk factors for SMI and investigate the impact of midday napping duration and depressive symptoms on SMI in older people, with the goal of uncovering potential interaction effects and provide insights for early intervention strategies to mitigate the risk of SMI. To achieve this, we analyzed representative cross-sectional survey data from China to assess the influences of midday napping duration and depressive symptoms on SMI among older people and explore potential underlying interaction effects.

## Methods

### Design and sample

We used the China Health and Retirement Longitudinal Study (CHARLS) data from 2018, which are publicly available at http://charls.pku.edu.cn. These survey data cover 28 provinces in China and contain information on individual factors, lifestyles, and health status of respondents. The data are of a high quality and have the nature of a large sample, thus providing real and effective data support for the analysis in this paper. Based on the purpose and needs of this study, the sample was screened, and 8,254 microsamples were obtained after excluding those with missing values of key variables. Our research was performed in accordance with the Declaration of Helsinki. The original CHARLS project was approved by the ethical review committee of Peking University (IRB00001052–11,015), and all participants signed an informed consent form at the time of enrollment.

**Fig. 1:**
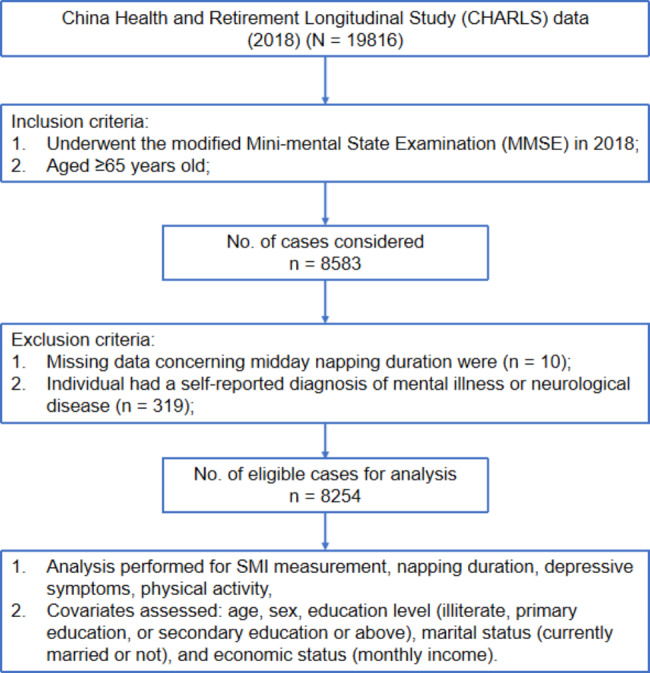
Flow chart of this study

Individuals initially eligible for participation were those who (1) underwent the modified Mini-mental State Examination (MMSE) in 2018 and (2) were aged ≥ 65 years old. MMSE is a widely used cognitive screening tool that evaluates various cognitive domains, including orientation, memory, attention and computation, and language. As previously reported, the selected cutoff scores for our investigation were 17 points for illiterate individuals, 20 points for participants with ≤ 6 years of education, and 24 points for participants with > 6 years of education, and cognitive impairment was suggested when the participant had an MMSE score lower than the appropriate cutoff score according to their years of education [31]. Hence, a total of 8,583 subjects were included. Then, the following criteria were applied for exclusion purposes: (1) data concerning midday napping duration were missing (n = 10) and (2) the individual had a self-reported diagnosis of mental illness or neurological disease (n = 319). Finally, we identified 8,254 subjects for our analysis. Figure 1 presents the flow chart of this study.

### Measurement of SMI

SMI refers to conditions in which people complain of memory problems despite intact cognition. The respondents were asked to respond to the question “how do you feel your memory is now?” by stating excellent, very good, good or fair, or poor. Then, the MMSE was used to measure the cognitive function of all older people participants. Subjects who complained of poor memory and who showed a normal MMSE test score were considered to have SMI [4, 32].

### Measurement of Napping Duration

The sleep variable was investigated by examining data on post-lunch napping, which were collected by asking the following question during the face-to-face interview: “during the past month, for how long (in minutes) did you take a nap after lunch?” Respondents were categorized according to their answer into short nap (0–30 min), moderate-length nap (31–90 min), and extended-length nap (> 90 min) groups. The cutoff values for napping time were chosen according to a previous study [33], and our study used a moderate-length nap time (31–90 min) as the reference value for nap time [21].

### Measurement of depressive symptoms

Depressive symptoms were assessed using the 10-item Center for Epidemiological Studies Depression Scale, which has been highly validated for use in general populations with adequate reliability and validity confirmed for the assessment of the community-dwelling older population in China. The respondents were asked to rate “how often you felt this way during the past week,” with total scores ranging from 0 to 30 points and a lower score indicating a lower level of depressive symptoms. A score of ≥ 10 points was used to identify the respondents who had significant depressive symptoms [23].

### Measurement of physical activity

The CHARLS questionnaire investigated the physical activity of participants in the preceding week. Participants were asked to indicate both the number of days that they performed ≥ 10 min of physical activity and the duration of their daily activity. We then estimated the physical activity energy expenditure or metabolic equivalent (MET) for 1 week using the following equation: MET-mins = MET level activity time (60 min) × number of activities per week. Then, we used the International Physical Activity Questionnaire to gather information on the frequency, duration, and intensity of physical activity performed by the participants in the week preceding the survey. The physical activity level was divided into low-intensity physical activity (< 600 METs/week), medium-intensity physical activity (600–3000 METs/week), and high-intensity physical activity (> 3000 METs/week). We used a moderate-intensity physical activity level as the criterion for adequate physical activity according to expert consensus [14, 34].

### Covariates

The covariates included in the analysis were sociodemographic characteristics such as age, sex, education level (illiterate, primary education, or secondary education or above), marital status (currently married or not), and economic status (monthly income).

Regarding education level, we obtained information through the question, “What’s [Name of the family respondent]’s highest achieved education?“ Respondents could indicate various levels, such as no formal education (illiterate), incomplete elementary schooling, sishu/home schooling, elementary school, middle school, high school, vocational school, two-/three-year college/associate degree, four-year college/bachelor’s degree, post-graduate, master’s degree, or doctoral degree/Ph.D. For analysis purposes, we classified them into three groups: illiterate, primary education, and secondary education or above. To determine marital status, we inquired, “What’s current marital status?“ The respondents had the option to select from the following choices: married with spouse present, married but temporarily not living with spouse due to reasons such as work, separated, divorced, widowed, or never married. We subsequently consolidated them into two categories: married and unmarried. As for economic status, we ascertained the monthly salary from the workplace, encompassing all bonuses, by asking, “What is your monthly salary from that workplace, including all bonuses?“ We then categorized the responses into three groups: <5000, 5000–10,000, and > 10,000.

### Statistical analysis

Data analyses were performed using Stata version 26 (STATA Corp., College Station, TX, USA). A descriptive statistical analysis was used to demonstrate the status of the sample, and the chi-squared test was used for univariate analysis. Logistic regression models were used to analyze the factors affecting SMI. Additive and multiplicative models were used to analyze the interaction effect of midday napping duration and depressive symptoms on SMI [35, 36]. The relative excess risk due to interaction (RERI), the attributable proportion (AP) due to interaction, and the interaction index (i.e., the synergy index) (S) estimates and 95% confidence interval (CI) values were calculated. Rothman suggests that if there is no additive interaction between the two factors, then the confidence interval of RERI and AP should contain 0, and the confidence interval of S should contain 1 [35]. A 2-tailed *P* value of < 0.05 was considered to indicate statistical significance.

## Results

### Participant characteristics

As shown in Table 1, more than half of the 8254 older people included in this study were female (n = 4240). The average (mean ± standard deviation) age of participants was 68.5 ± 6.5 years old, and the 65–74 age group contained the largest proportion of participants (80.9%). A total of 4401 participants were illiterate (53.3%), 6642 (80.4%) were married, and the most common monthly income was 5000 (67.5%). Among the respondents, 5280 (63.9%) had SMI. A total of 3785 (45.86%) took short naps (0–30 min), while 3210 (38.89%) took moderate-length naps (31–90 min), and 1259 (15.25%) took extended-length naps (> 90 min). A total of 3206 (38.8%) participants had depressive symptoms.

Univariate analysis detected significant differences in gender, age, education level, marital status, economic status, depressive symptoms, midday napping duration, and physical activity between the non-SMI and SMI groups (P < 0.05). Detailed results are shown in Table 1.

**Table 1:** Definition, descriptive of variable

Variable	Definition	mean ± sd	n (%)	χ_2_	P
Gender	Male = 1, female = 0.	0.49 ± 0.50	Female = 4240(51.37) Male = 4014(48.63)	34.05	< 0.01
Age group (years)	65–74 years old = 1, 75–84 years old = 2,≥ 85 years old = 3.	1.21 ± 0.45	65–74 = 6685(80.99) 75–84 = 1408(17.06) ≥ 85 = 161(1.95)	350.22	< 0.01
Educational level	Illiterate = 1, Primary school = 2, Middle school or above = 3.	1.71 ± 0.84	Illiterate = 4401(53.32) Primary = 1808(21.9) Middle = 2045(24.78)	27.15	< 0.01
Monthly income (RMB)	< 5000 = 1, 5000–10,000 = 2, > 10,000 = 3.	1.60 ± 0.89	< 5000 = 5578(67.58) 5000 ~ 10,000 = 411(4.98) > 10,000 = 2265(27.44)	60.24	< 0.01
Marital status	Married = 1, Unmarried = 0.	0.80 ± 0.40	Married = 6642(80.47) Unmarried = 1612(19.53)	130.73	< 0.01
Physical activity	Low intensity = 1, Medium intensity = 2, High intensity = 3	1.92 ± 0.56	Low = 1690(20.47) Medium = 5575(67.54) High = 989(11.98)	35.87	< 0.01
Midday napping duration	Midday’s napping duration time, 0–30 min = 1, 31–90 min = 2, > 90 min = 3.	2.23 ± 1.11	0-30 min = 3785(45.86) 30-90 min = 3210 (38.89) > 90 min = 1259(15.25)	14.90	< 0.01
Depressive symptoms	According to the question of DC009-DC018 in the questionnaire, to sum up all, the range of the score is 0–30, and the people whose score is above 10 is under depressive symptoms, Yes = 1, No = 0	0.39 ± 0.49	Yes = 3206(38.84) No = 5048(61.16)	9.38	0.002
MMSE scores	MMSE is a widely used cognitive screening tool that evaluates various cognitive domains, including orientation, memory, attention and computation, and language.	17.75 ± 5.93			

*Note*: Categorical variables are presented as frequencies with percentages, n(%) represents the number of each sample and its percentage, SMI represents subjective memory impairment, RMB represents Chinese Yuan, min is minutes, P is the test level

### Status of depressive symptoms, midday Napping Duration, and incidence of SMI

Among the respondents, 5280 (63.9%) had SMI. A total of 3785 (45.86%) took short naps (0–30 min), while 3210 took moderate-length naps and 1259 (15.25%) took extended-length naps (> 90 min). A total of 3206 (38.8%) participants had depressive symptoms. Univariate analysis detected significant differences in gender, age, education level, marital status, economic status, depressive symptoms, midday napping duration, and physical activity between the non-SMI and SMI groups (*P* < 0.05). Detailed results are shown in Table 1.

### Affecting factors of SMI

Multi-factor logistic regression analysis showed that gender (odds ratio [OR] = 1.49), age (OR = 2.05, 3.71), education (OR = 0.62, 1.99), marital status (OR = 0.73), economic status (OR = 0.70, 0.52), depressive symptoms (OR = 1.29), midday napping duration (OR = 1.27, 1.52), and physical activity (OR = 0.82, 0.99) were significant factors affecting SMI in older people. More details can be found in Table 2.

**Table 2:** Logistic regression analysis of SMI in Chinese older people

Variable	OR (95%CI)	*S*	*Z*
**Age (years)** (ref = 65–74 years)			
75–84	2.03 (1.76,2.33)	0.14	9.91
≥ 85	3.70 (2.32,5.88)	0.87	5.54
**Gender** (ref = male)			
Female	1.50 (1.36,1.66)	0.07	7.93
**Marital status** (ref = unmarried)			
Married	0.73 (0.64,0.84)	0.04	−4.59
**Educational level** (ref = illiterate)			
Primary school	0.62 (0.55,0.70)	0.03	−7.73
Middle school or above	2.03 (1.77,2.32)	0.13	10.40
**Economic situation** (ref = < 5000RMB/monthly)			
5000–10,000	0.70 (0.56,0.86)	0.07	−3.30
> 10,000	0.52 (0.46,0.59)	0.03	−10.4
**Physical activity** (ref = low intensity) (ref = Low intensity)			
Medium intensity	0.83 (0.73,0.94)	0.05	−2.93
High intensity	0.99 (0.83,1.19)	0.09	−0.04
**Depressive symptoms** (ref = no)			
Yes	1.29 (1.17,1.44)	0.07	4.87
**Midday napping duration** (ref = 31-90 min) duration30-90 min)			
Short nap (0–30 min)	1.27 (1.14,1.40)	0.06	4.60
Extended-length nap (> 90 min)	1.52 (1.31,1.76)	0.11	5.69

*Note*: SMI is subjective memory impairment, RMB is the Chinese Yuan, min stands for minutes, S is the standard error, and Z is the regression coefficient divided by its standard error

### Multiplicative Interaction Effect of depressive symptoms and midday napping duration on SMI

A multiplicative model [35] was used to analyze the interaction effect of midday napping duration and depressive symptoms on SMI. After controlling the confounding factors, there was no multiplicative interaction effect between depressive symptoms and short nap or extended-length nap on the occurrence of SMI in older people (*P* > 0.05). Detailed results are shown in Table 3.

**Table 3:** Multiplicative interaction effect of midday napping duration and depressive symptoms on the incidence of SMI in older people

Variable	OR	*S*	*Z*	*P*	95% CI	
Short nap (0–30 min)	1.26	0.08	3.60	< 0.01	1.11	1.43
Extended-length nap (> 90 min)	1.57	0.14	4.94	< 0.01	1.31	1.87
Depressive symptoms	1.30	0.10	3.31	< 0.01	1.11	1.53
Short nap * depressive symptoms	1.01	0.10	0.09	0.92	0.81	1.24
Extended-length nap * depressive symptoms	0.91	0.14	−0.54	0.58	0.67	1.24

*Note*: The model was adjusted for variables of gender, age, education, marital status, and economic status. Subjects without depressive symptoms and moderate-length naps was considered as the reference group. SMI is subjective memory impairment, min represents minutes, S is the standard error, Z is the regression coefficient divided by its standard error, P is the test level, CI is the confidence interval, and CI is the 95% CI.

### Additive Interaction Effect of depressive symptoms and midday napping duration on SMI

An additive model was used to analyze the interaction effect of midday napping duration and depressive symptoms on SMI in older participants. We designated those without depressive symptoms, who also took moderate-length naps, as the reference group. This study presented the cross-classification of midday napping duration and depressive symptoms and their relationship with SMI. In cases where there is a midday napping duration of 0–30 min and no occurrence of depressive symptoms, the risk of SMI is 1.2 times higher than in cases with a midday napping duration of 30–90 min and the presence of depressive symptoms. Please refer to Table 4 for details. Our analysis identified a positive additive interaction effect between the presence of depressive symptoms and both short and extended-length naps. When both extended-length naps and depressive symptoms were present in an older individual, the risk of SMI was 1.06 times higher than when either extended-length naps or depressive symptoms were observed individually (RERI = 0.27, 95% CI = 0.07–0.43; AP = 0.14 95% CI = 0.01–0.23,S = 1.06, 95% CI = 0.79–1.82). Similarly, the co-occurrence of short naps and depressive symptoms elevated the risk of SMI by 1.2 times in comparison to the presence of short naps or depressive symptoms in isolation (RERI = 0.12, 95% CI = − 0.14 to 0.39; AP = 0.13, 95% CI = − 0.07 to 0.22, S = 1.20, 95% CI = 0.57–1.62). The respective AP values of 0.13 and 0.14 indicate that the interactive effect of depressive symptoms and short naps accounted for 13% of the SMI cases in our study population. Meanwhile, the interaction of depressive symptoms and extended-length naps were responsible for 14% of the SMI cases. For a more detailed overview, please refer to Tables 4 and 5.

**Table 4:** Additive interaction of different napping durations (short naps or extended-length naps), depressive symptoms, and SMI in older participants

Different napping duration		Depressive symptoms	OR	*S*	*Z*	*P*	95% CI	
Short nap (0–30 min)	Yes	No	1.20	0.06	3.29	< 0.01	1.07	1.35
	No	Yes	1.35	0.07	5.27	< 0.01	1.21	1.52
	Yes	Yes	0.84	0.07	−1.99	0.04	0.71	0.99
Extended-length nap (> 90 min)	Yes	No	1.41	0.10	4.82	< 0.01	1.23	1.63
	No	Yes	1.40	0.08	5.62	< 0.01	1.24	1.57
	Yes	Yes	0.77	0.07	−2.50	< 0.01	0.63	0.94

*Note*: SMI is subjective memory impairment, S is the standard error, Z is the regression coefficient divided by its standard error, P is the test level, CI is the confidence interval, and CI is the 95% CI.

**Table 5:** Estimated value of the additive interaction effect

	Short nap	Extended-length nap
RERI	0.12 (− 0.14–0.39)	0.27 (0.07–0.43)
AP	0.13 (− 0.07–0.22)	0.14 (0.01–0.23)
S	1.20 (0.79–1.82)	1.06 (0.57–1.62)

*Note*: AP, attributable proportion due to interaction; CI, confidence interval; RERI, relative excess risk due to interaction; S, synergy index

## Discussion

The present study’s findings revealed an SMI incidence of 63.9% among the older participants, significantly surpassing the rates of a prior study involving community-dwelling older individuals in Korea (22%) [37], as well as Tobiansky R’s discoveries (25%). Nevertheless, this figure remained lower than the prevalence of SMI reported in another study conducted among registrants aged above 65 years in two primary care services in south London (66.7%) [9]. Such variations may be attributed to the disparate screening tools employed for SMI assessment. Kim and colleagues utilized the Geriatric Mental State Scale to evaluate memory function, whereas Tobiansky and associates employed a semi-structured interview known as short-CARE. In the present study, SMI screening relied on a single subjective question, inquiring whether older people felt memory decline, alongside the MMSE. Comparatively, the SMI incidence in the current study remained lower than that (70%) reported by another study [32], which involved respondents from rural areas with lower levels of education and economic status. This earlier study’s findings suggested that SMI is indeed highly prevalent among older people. Our hypothesis posits that the observed disparities in objective memory test results, when compared to previous studies, could be attributed to varying incidence rates of SMI within the populations studied. Factors influencing these differences could range from unique regional, social, and cultural contexts to the specific screening tools employed in these studies.

Subjective memory impairment refers to an individual’s self-perception of memory decline or difficulties, despite the absence of objective cognitive impairment discernible through standardized tests [38]. In research centered on objective memory tests, self-reported measures of SMI often serve as a critical tool for identifying individuals who may have concerns about their memory. However, the inclusion criteria for SMI can vary across studies. Some studies may have a broader definition of SMI, leading to a higher incidence of individuals reporting memory problems. On the other hand, studies with stricter criteria may have a lower incidence of SMI. The variation in SMI incidence can impact the composition of study samples, potentially introducing heterogeneity in objective memory test results. Higher SMI incidence may include individuals with different underlying factors contributing to memory complaints, such as depression, anxiety, or other health conditions [39, 40]. This diversity in the etiology of SMI could affect cognitive performance, leading to inconsistent objective memory test outcomes across studies. To address these issues, community healthcare personnel should pay closer attention to older people with SMI, adopt more sensitive screening tools for early detection, enhance the regularity of assessments to identify memory changes, and provide memory or cognitive training as early as possible to protect the cognitive function of older people.

The results of the current study revealed that older people with SMI tend to have certain demographic factors, including older age, female sex, an unmarried status, and a lower economic status (monthly income), which are consistent with the findings of previous studies [7, 41, 42]. This study also found that older people with a high level of education (secondary or above) were more likely to report SMI, while previous studies have suggested that high education is a protective factor of memory in older people [43, 44]. Whereas there was also evidence that the risk of Alzheimer’s disease associated with subjective memory complaints is higher among those with higher education than those with lower education [45]. Older people with elevated education levels tend to access to obtain more health knowledge [46], who are more aware of their health problems and sensitive to memory impairments, and thus are more likely to provide external feedback on their memory impairment problems. Especially in highly educated individuals, who still perform well on formal cognitive tests, subjective memory impairment may be an important first sign of impending Alzheimer’s disease [45]. Therefore, it becomes crucial to monitor whether older individuals with a higher level of education present complaints or behaviors indicative of memory problems. Additionally, there is a need to develop more accurate screening tools for early identification of SMI, thereby avoiding any potential under-detection.

This study also found that midday napping duration is one of the important factors influencing the occurrence of SMI. Short naps (0–30 min) and extended-length naps (> 90 min) are greater risk factors for SMI compared to moderate-length naps (31–90 min). Previous studies have also found that moderate-length naps benefit memory and cognitive function in older people [47]. Blackwell and associates further indicated that the longer the midday napping duration is, the higher the risks of memory decline and cognitive impairment [16]. Extended-length naps affect the occurrence of SMI in older people by influencing the sleep/wake circadian rhythm and disrupting nighttime sleep, leading to impairments in memory and cognitive function [48]. Prior research likewise focused on the relation of midday napping duration and cognitive performance, with mixed findings in different studies [49, 50]. As a consequence, more epidemiological studies are needed to confirm this association. Midday napping represents a cost-effective approach with the potential to safeguard memory function, making it highly suitable for widespread implementation among the population. Community health care professionals should proactively offer expert guidance to older people who either refrain from napping or opt for brief or prolonged naps, aiding them in cultivating the beneficial practice of indulging in moderate-length naps.

We further found that depressive symptoms are another important factor influencing the occurrence of SMI in older people, which is supported by previous studies [36, 47, 51, 52]. For example, a cross-sectional study of community adults indicated that the occurrence of SMI was strongly associated with depressive symptoms [53]. Another study further revealed that higher levels of depression were independently associated with more severe memory impairment [54]. Several mechanisms can explain the associations between depressive symptoms and SMI. First, older people with depressive symptoms tend to evaluate their memory performance negatively and to amplify their memory impairment [55]. Second, patients with depression have higher levels of inflammatory cytokines, and elevated inflammatory markers are associated with poorer memory and cognitive function [56, 57]. Most mechanistic studies have mostly focused on groups with MCI; however, SMI is a precursor state of MCI, and more studies focusing on the mechanism by which depressive symptoms relate to SMI are warranted.

It is worth emphasizing that short naps, extended-length naps, and depressive symptoms had positive additive interaction effects, respectively, on the risk of SMI in older people. Our study showed that the interaction effect between short naps and depressive symptoms and that between extended-length naps and depressive symptoms are more detrimental than moderate-length naps to memory and cognitive function in older people. Wang and associates found that subjects who do not nap had higher risks for memory and cognitive impairments than those who take moderate-length naps [47]. Healthcare professionals may consider incorporating structured nap interventions as part of the treatment plan for individuals with depression, which could involve recommending specific nap durations, timing, or sleep hygiene practices to optimize the beneficial effects [58].

Compared to the above mentioned study, approximately 45% of participants in this study had a 0-30 min short nap in the current study. It has been reported that insufficient napping duration is more likely to promote neuroinflammation and nerve destruction, particularly in the hippocampus, a key neuroanatomical region of memory, leading to neurodegenerative degeneration [59]. However, the mechanisms of the interaction effect between different napping duration and depressive symptoms on the occurrence of SMI in older people have not yet been determined. In the future, more empirical studies are needed to explore the dose–effect relationship between midday napping and SMI. Community health professionals could develop effective interventions to relieve depressive symptoms and help patients form regular midday napping habits; for example, in a previous study, a homemade gravity blanket was provided to depressive disorder co-morbid insomnia patients to influence adjustments to their nap time in order to reduce depressive symptoms [60].

This study also revealed the pivotal role of physical activity in influencing the occurrence of SMI among older people. Notably, older people who participate in medium- to high-level intensity physical activities display enhanced memory capacities, a finding corroborated by previous research indicating that the overall volume of physical activity significantly impacts cognitive function in older people [61–63]. Furthermore, another study further indicated that moderate or above physical activity for more than 90 min during the week slowed the rate of cognitive decline in older adults and contributed to the improvement of cognitive function [64]. The mechanism by which physical activity benefits memory and cognitive function can be explained according to physiological and psychological aspects. First, physical activity improves cerebral blood flow and cerebral perfusion and thus has a positive effect on brain memory function [65]. Second, people who engage in outdoor group physical activities tend to have more social links and social roles, which increase their sources of social support and reduce social isolation.

However, it should also be noted that high intensity physical activity did not show a significant protective effect against SMI in older people in this study, which could be attributed to several factors. First, it could be possible that the specific group of older people included in this study had relatively similar levels of physical activity, with limited variability in the intensity of their physical activities, whereby if the majority of participants engaged in moderate to high intensity physical activities, this might diminish the ability to detect a protective effect specifically for high intensity physical activity. Second, SMI can be influenced by various factors, including age, gender, education level, depressive symptoms, and economic status, as shown in the logistic regression analysis. It is possible that these factors have stronger associations with SMI in this particular study population compared to the intensity of physical activity. Lastly, from an overall perspective, we believe that the relationship between physical activity and cognitive function is multifaceted and can be influenced by various factors, such as genetic predispositions, overall health status, and duration of physical activity engagement. High intensity physical activity might have different effects on cognitive outcomes depending on these individual factors, making it challenging to identify a consistent protective effect against SMI. Thus, more research is needed to draw definitive conclusions regarding the relationship between different level of physical activity intensity and SMI.

Several enhancements can be implemented to fortify this study. Firstly, utilizing cross-sectional data from CHARLS presents a challenge in inferring cause-and-effect relationships between SMI, depressive symptoms, and midday napping duration. To establish a temporal relationship between SMI and the primary influencing factors, future longitudinal studies should be conducted. Secondly, the assessment of midday napping duration in this investigation relied on respondents’ subjective judgments, which could potentially lead to misreporting and introduce information bias. To mitigate this concern, future research could incorporate objective tools to accurately record nap times. Thirdly, future studies should do cohorts to explore causal relationships between midday napping duration, depression and SMI. Lastly, MMSE has its limitations, particularly in populations with higher education levels where it may be less sensitive to detect cognitive impairments. To address this concern and increase the accuracy of cognitive assessment, future studies could consider incorporating additional neuropsychological tests that are more sensitive to subtle cognitive changes in individuals with higher education levels.

## Conclusion

Midday napping duration (Short naps and extended-length naps) and depressive symptoms had positive additive interaction effects on the occurrence of SMI in older people. Based on our study, we would more than recommend medium-time naps. Community healthcare worker can reduce risk of SMI by helping older people to maintain healthier napping habits, and teaching older people and their families to detect and treat depressive symptoms effectively.

## Data Availability

The datasets generated and/or analysed during the current study are available in the [the China Health and Retirement Longitudinal Study (CHARLS)] repository, [http://charls.pku.edu.cn].
